# Occurrence of Microplastics and Cytotoxic Effects of Organic Extracts from Isolated Mesoplastics in Compost

**DOI:** 10.1021/acsomega.6c01919

**Published:** 2026-05-19

**Authors:** Paloma Sánchez-Argüello, Gema Sáez-Salto, Alice Budai, Pierre A. Rivier, Simon Weldon, Antonio Martín-Esteban

**Affiliations:** 1 Departamento de Medio Ambiente y Agronomía, 16379 INIA-CSIC, Carretera de A Coruña km. 7.5,Madrid 28040, Spain; 2 Division of Environment and Natural Resources, Norwegian Institute of Bioeconomy Research (NIBIO), Ho̷gskoleveien 7, Ås 1432, Norway

## Abstract

Compost application is a widely recommended practice to maintain and improve soil fertility. However, such a practice could be a main entry path for plastic into soil. Accordingly, in the present work, two different compost samples, obtained with and without biochar, were analyzed to investigate how composting can affect the presence of microplastics (MPs). The substrate of both samples (consisting of a mixture of household food waste and animal manure) was also analyzed for comparative purposes. Samples were processed by oxidation, flotation, and filtration. MPs on the filters were observed, counted, and size-calibrated using both a stereomicroscope and an inverted microscope. MPs larger than 1 mm were further characterized by attenuated total reflectance Fourier-transformed infrared spectroscopy (ATR-FTIR). In parallel, mesoplastics (0.5–2 cm) were recovered from substrate and compost and extracted in methanol for testing *in vitro* cytotoxicity. The estimated concentration of MPs ranged from 820 to 1340 fragments/kg of dry sample, depending upon the sample. Three polymers represented the totality of identified plastic items: polyethylene (PE, including both low and high density), polyethylene terephthalate (PET), and polypropylene (PP) in order of abundance. Nevertheless, cytotoxicity was only observed in mesoplastic extracts from the substrate and could not be attributed to the identified plastic items themselves, suggesting that cytotoxic effects could have been caused by contaminants adsorbed to plastics or by the leaching of plastic additives during the extraction process. In summary, the composting process reduced the cytotoxicity of plastic extracts and the presence of MPs in compost, which could be attributed to the fragmentation of plastics.

## Graphical abstract

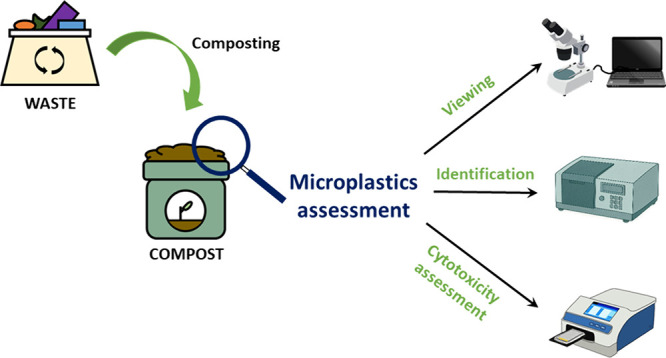

## 1 Introduction

Today, microplastics (MPs) are considered one of the main contaminants of the different environmental compartments including air, water, and soil and thus have been the subject of intensive study during the past decade.^1–5^ MPs are plastic fragments with a size less than 5 mm in diameter. This small size allows MPs to be dispersed in the environment and enter the food chain^6,7^ and/or drinking water,^8^ which represents a still unknown risk to human health.^9,10^ Most previous studies have focused on understanding the distribution and fate of MPs in freshwater and marine environments. However, despite the existence of studies reporting that there may be 4 to 23 times more MPs on land than in the ocean,^11^ very little information can be found on the diversity of MPs present in soils and their potential impact on soil fertility,^12^ which is especially relevant for agricultural soils. Although MP contamination of agricultural soils is considered to be ubiquitous, the application of sewage sludge and compost as organic fertilizers represents an important source of plastic contamination among others.^13–16^ Sources of MPs in compost are diverse. Sewage sludge can retain polyester, nylon, and acrylic microfibers from household sources, such as washing clothes, as well as MPs contained in personal care products. Household food waste can also contain residue from plastic bags, which can be integrated into composting processes. Finally, the use of biosolid residues from livestock farming also contributes to MP contamination in composting, since livestock can ingest MPs from contaminated feed and subsequently excrete them in their feces. In this regard, it has been reported that considering common recommendations in composting practice, application of 7 to 35 tons of compost ha^–1^ to agricultural fields leads to plastic loads of 84,000 to 1,610,000 plastic items ha^–1^ per year in Germany,^13^ demonstrating the importance of compost application as a source of MP contamination. The amount and the properties (i.e., size, fragility) of MPs present in compost depend upon the corresponding feedstocks and composting experimental conditions.^17–19^ For instance, in several aerobic decomposition studies, plastics fragmentation was observed leading to a relevant increase in the number of particles,^20,21^ whereas the hydrolysis process during anaerobic fermenting resulted in the brittleness of plastics.^22^ In this regard, aerobic composting is considered to be an excellent (environmentally friendly and cost-effective) technology for removing MPs from organic waste and improving their biodegradability.^23^ For instance, Xing et al. reported a 35.93% reduction in MP abundance and a significant decrease in particle size after composting.^24^ Accordingly, the effect of feedstocks and composting experimental conditions on the presence of MPs in the resulting compost is worthy of investigation, as described in detail by Dong et al.^25^ In this regard, one of the approaches studied to enhanced MP degradation was the use of biochar.

Biochar, which can be produced through the pyrolysis of waste biomass, has shown to improve the composting process through reductions in greenhouse gas emissions,^26^ reductions in maturation time, and increasing temperature development and stability.^27–29^ Besides, biochar can have a positive effect on the final compost product, increasing its value as a fertilizer through positive effects on plant yields and nutrient retention.^30^ Sun and coauthors^31^ demonstrated that adding livestock manure biochar to compost increased the degradation of MPs compared to controls without biochar. Similarly, the present work examines the presence of MPs in compost with and without the addition of biochar during processing. A substrate, consisting of a mix of household food waste, industrial waste, and manure, was used as feedstock for composting and the presence of MPs was also analyzed for comparative purposes. In parallel, we investigated the toxicity of organic extracts of mesoplastics present in compost as plastics can accumulate lipophilic organic pollutants. According to previous studies, the use of *in vitro* cytotoxicity is a suitable approach for investigating the toxicity of pollutants sorbed onto plastics.^32^

This study combines techniques to identify and quantify MPs, together with and *in vitro* assessment of the cytotoxicity of organic extracts from mesoplastics, in order to evaluate the effects of compost processing, including the addition of biochar, on the quality of compost from the perspective of plastic presence. Plastics in compost have undergone environmental aging and transformation, and the potential toxic effect of these complex materials—arising from a combination of polymer properties, additives, and sorbed pollutants—are important to consider in compost quality characterization. Fish cell-based toxicity assays were selected as an additional rapid screening tool requiring very small sample volumes (i.e., organic extracts), which can indicate potential aquatic hazards associated with compost-amended soils through the leaching of complex mixtures of pollutants associated with plastics. This integrated approach could be useful in developing a standardized methodology for assessing the impact of plastics on compost quality.

## 2 Experimental Section

### 2.1 Reagents

Minimal essential medium (MEM), fetal bovine serum (FBS), l-glutamine, MEM nonessential amino acids, penicillin/streptomycin, and trypsin-EDTA for cell culture were supplied by Biowest-Labclinics (Spain). Neutral red (NR) was purchased from Sigma-Aldrich (Germany).

A Milli-Q water purification system from Millipore (Bedford, MA, USA) provided deionized water for this study. All other reagents used were analytical grade.

### 2.2 Samples Analyzed

The substrate (dry matter 14%) employed for composting consisted of a mix of 49% household food waste, 5% industrial waste (blood, fat, etc.), and 46% manure (consisting of 54% cow manure, 32% of pig manure, and the rest a mix of cow and pig manure). The biochar (from Pyreg) consisted of mixed wood pyrolyzed at 550 °C HTT (highest treatment temperature). It was added based on dry weight (5% dry weight of the total mixture), and it corresponded to 3 L in volume. This volume was compensated in the treatment without biochar by adding 3 L of wood shavings to it. Wood shavings were used for increasing C/N ratio, and wood chips were used for structural purposes. The resulting starting mixtures for compost 1 and compost 2 had a C/N ratio of 24, while the compost products had a C/N ratio of 19 after maturation.

There were six composts produced (two treatments, three replicates) made of substrate composted without (compost 1) and with (compost 2) biochar. Composts made from substrate consisted of a mix of substrate (30 L), wood shavings (65 L), and wood chips (20 L). Composts were turned daily during the thermophilic stage (3 weeks above 50 °C with peaks above 65 °C) and then turned every second week during the maturation phase (about 6 months).

### 2.3 Microplastics Analysis

To assess the presence of MPs in selected samples, a filtration/oxidation/flotation procedure was adapted from elsewhere.^13^ Briefly, 5 g of dry samples was sequentially sieved through 2, 0.8, 0.45, and 0.2 mm sieves. MP fragments larger than 0.8 mm were collected with microtweezers, observed using a stereomicroscope and subjected to infrared spectroscopy analysis as described below. Sample fractions below 0.8 mm were collected and placed in three different vessels and treated independently with 10 mL of 33% H_2_O_2_ for 24 h. Then, 100 mL of ZnCl_2_ solution (saturated, density = 1.6 g mL^–1^) was added, stirred for 1.5 h, and allowed to stand for 2 h for particle sedimentation. Finally, liquid extracts were sequentially filtered three times through 20–25 μm Whatman paper filters (Cytiva, Buckinghamshire, UK) and stored for further analysis.

To avoid contamination, all materials used in the sample preparation process were rinsed with Milli-Q water and dried and covered with aluminum foil before the experiments. All liquids were filtered through a 0.22 mm filter paper prior to use. The whole-sample preparation process was performed in a clean fume hood to prevent contamination with airborne MPs. By strict adherence to these measurements, no contamination from materials or blank reagents was observed.

MPs on the filters were first observed visually and counted using a stereomicroscope. Those MPs large enough to be separated from the filter with microtweezers were stored for further observation using an inverted microscope equipped with a digital camera for size calibration. Those MPs larger than 2 mm were further characterized by Fourier transform infrared spectroscopy (FTIR) as described below. Besides, collected MPs were subjected to cytotoxicity studies. A flowchart summarizing the whole experimental procedure is displayed in Figure 1.

**Figure 1:**
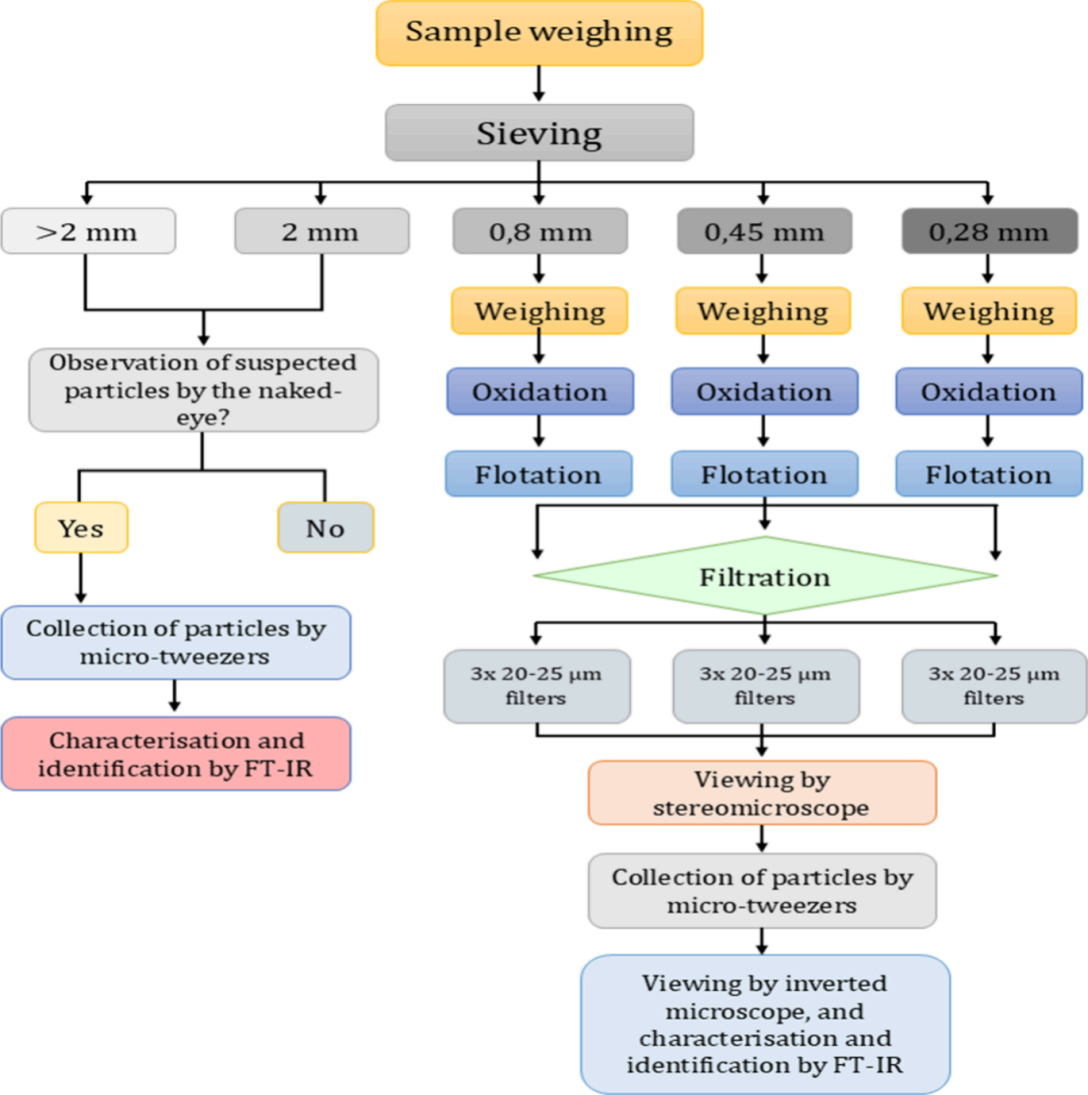
Flow chart describing the whole experimental procedure for the isolation of microplastics from compost samples.

### 2.4 Fourier-Transformed Infrared Spectroscopy (FTIR) Analysis

As mentioned above, those MPs larger than 1 mm were further characterized by Fourier-transformed infrared spectroscopy (FTIR) on a Jasco FTIR-460 Plus spectrophotometer (Madrid, Spain). All spectra were recorded by attenuated total reflectance (ATR) between 3600 and 600 cm^–1^. MPs were identified by comparing FTIR spectra with open-access databases,^33^ and with our own database, using the optical spectroscopy software Spekwin 32.^34^ After comparison, MP identification was considered successful when the matching score was higher than 70%.^35^

### 2.5 Mesoplastic Cytotoxicity Testing

Mesoplastics (0.5–2 cm) were collected and used without pretreatment to extract all the potential toxic components both inherent and absorbed. Mesoplastics from the different samples (substrate, compost 1, and compost 2) were extracted with methanol as described elsewhere.^36^ Briefly, all visible mesoplastics in 1 kg of sample were collected. A total of 0.2 g mesoplastics in substrate and compost 1 and 0.15 g in compost 2 were extracted with methanol in a 1:10 ratio (w/v) in an ultrasound bath for 1 h, followed by a second extraction in the same conditions. Extracts were mixed and centrifuged at 2500 rpm for 5 min. Liquid fractions were collected and evaporated to dryness under a gentle nitrogen stream. The obtained residues were finally reconstituted in 100 μL (or 75 μL for compost 2) of dimethyl sulfoxide (DMSO), which corresponded to 2 g/mL of mesoplastic equivalents in DMSO, and stored at 4 °C for further experiments.

In parallel, sample extracts were prepared from a food packaging film, a mineral water bottle, and drinking straws, which are mainly composed of polyethylene (PE), polyethylene terephthalate (PET), and polypropylene (PP), respectively. The same extraction procedure was used for these consumer plastic products, and the observed cytotoxicity was compared with that of mesoplastics. These materials were used as reference plastics since they mainly contain PE, PET, and PP, the plastic polymers found in the substrate and compost samples (Figure 4). However, considering that the substrate for composting consisted of 49% household food waste (see Section 2.2), using these reference plastics helps to create a simulation of realistic exposure conditions, as plastics found in compost derived from household waste are more likely to originate from everyday consumer products (e.g., packaging materials, bottles, and disposable items). The aim of the cytotoxicity tests is to compare the effects of the selected reference plastics with mesoplastics in the compost, which are expected to be environmentally aged and composted household plastic materials.

The RTG-2 fish cell line (ATCC, CL No. 55) was used to evaluate the cytotoxicity of plastic extracts, as an indication of the potential aquatic hazard posed by the leaching of plastic materials present in the compost. RTG-2 is one of the oldest and best-characterized fish cell lines in ecotoxicology and is widely used as an alternative to *in vivo* fish testing. Culture conditions have been described elsewhere.^37^ For cytotoxicity testing, cells were seeded in 96-well microplates and allowed to attach under the same culture conditions (20 °C in 5% CO_2_ in air). The cell monolayer was allowed to grow exponentially for 24 h and then treated with mesoplastic and reference plastic extracts in minimal essential medium (MEM), supplemented with 10% fetal bovine serum (FBS), penicillin–streptomycin (50 U/mL and 50 μg/mL, respectively), 2 mM glutamine, and MEM nonessential amino acids. Each treatment was replicated eight times per plate. In addition, supplemented MEM was used as a growth medium control and 1% DMSO as a solvent control. After 24 h of cell exposure, the Neutral Red Uptake (NRU) assay was used for testing cytotoxicity. NRU assay is based on the uptake of the Neutral Red dye into the lysosomes of viable cells. The growth medium with or without treatments (plastic extracts from samples and from commercial plastics) was removed and replaced with 100 μL of growth medium containing 40 μg/mL NR per well, and cells were incubated for 3 h at 20 °C, followed by incubation with 50% ethanol/1% acetic acid for 30 min. Finally, the absorbance was measured at 550 nm using a GENios spectrophotometer plate reader (Tecan, Switzerland).

Mesoplastics extracts were tested in supplemented MEM at concentrations of 0.25, 0.5, and 1% (v/v). This means that cells were exposed to extracts corresponding to 5, 10, and 20 g mesoplastic equivalents per liter of MEM. The cellular response to plastic extracts was expressed as percentage of cell viability (i.e., absorbance produced by uptaken NR) relative to growth medium control since solvent control (1% DMSO) had no effect on cell viability.

## 3 Results and Discussion

### 3.1 Preliminary Studies and Microplastics Isolation Optimization

Before analyzing the target samples described in the Section 2 (substrate, compost 1, and compost 2), the presence of MPs was assessed in three different compost samples from previous studies following initially the procedure described by Braun et al.^13^ The analyzed samples were one MP-free compost sample from green waste (compost A); one compost sample together with its corresponding feedstocks (biogas digestate, garden waste, and biochar) (compost B);^29^ and one compost sample obtained from green waste, the organic fraction of municipal solid waste, and biochar (compost C).^38^ The mentioned samples were analyzed for comparative purposes and reoptimization of our own experimental microplastic analysis procedures.

Initially, about 200 g of samples was sequentially sieved through 2 and 0.8 mm sieves. Then, each sieved fraction was subjected to density separation using 400 mL of a saturated ZnCl_2_ solution (density = 1.6 g mL^–1^) as described elsewhere.^13^ However, such a single-separation procedure was unsuccessful, leading to the obtainment of cake-like solutions, making proper handling and filtration for the isolation of MPs impossible. Thus, in order to improve the experimental procedure, the initial sample amount was reduced and the density separation procedure tested again after each sample reduction. Only when the initial sample amount was reduced to 5 g was it possible to filter the solutions obtained after density separation. However, the amount of solid matter was still too large, making not only the filtration process difficult but also the subsequent MP isolation.

Accordingly, additional sieving steps were added through 0.45 and 0.2 mm sieves. In addition, although it has been reported that the introduction of an oxidation step to reduce the amount of organic matter in compost samples is not recommended due to the formation of foam,^13^ it was decided to include a H_2_O_2_ treatment in a 1:10 sample/H_2_O_2_ ratio (w/v) for 24 h under stirring before density separation. Following this procedure, it was possible to obtain rather clear solutions, which were properly filtered through three different 20–25 μm filters allowing microplastic isolation. As a summary, all the parameters studied and selected experimental conditions for microplastic particle extraction from compost samples are shown in Table 1 and a flow chart describing the whole experimental procedure is depicted in Figure 1.

**Table 1:** Parameters Studied and Selected Experimental Conditions for Microplastic Particle Extraction from Composts

parameter	range studied	selected experimental conditions
sample size (g)	5–200	5
sieves (mm)	2, 0.8, 0.45 and 0.28	sequential sieving trough all indicated sieves
oxidation (ratio sample/H_2_O_2_)	1:5; 1:10; 1:20	1:10

The abundance of MPs in the mentioned samples, following the optimized procedure described above, ranged as expected from 0 microplastic fragments in compost A to 5000 microplastic fragments per kg of dry sample in compost C. As an example, Figure 2 shows some fragments of different color, shape, and size, visually observed by a stereomicroscope (circle) and an inverted (square) microscope, isolated from the sample compost C.

**Figure 2:**
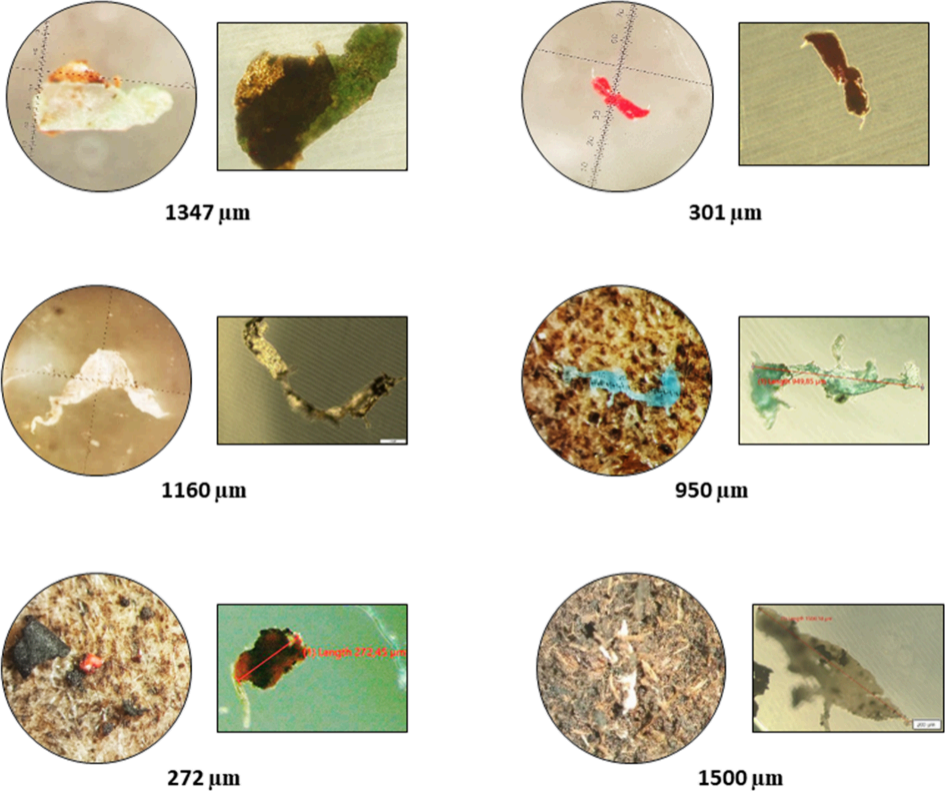
Fragments visually observed, by a stereomicroscope (circle) and an inverted (square) microscope, isolated from the sample compost C.

Such results agreed with the content of MPs determined in compost samples in other reported studies,^19,39,40^ which depends on the presence of MPs in compost feedstock. This presence varies considerably; for example, the number of MPs per kilogram ranges from 902 to 24,000 MP fragments per kilogram in swine manure and sewage sludge.^25^ Thus, the optimized procedure (Figure 1) was applied to the assessment of the presence of MPs in target samples of the present study (substrate, compost 1, and compost 2) described in Section 2.

### 3.2 Abundance, Size Distribution, and Identification of MPs

As mentioned above, target samples were subjected to the optimum experimental procedure for the isolation of MPs, and the changes in the abundance and size distribution of MPs in substrate and compost without (compost 1) and with biochar (compost 2) were observed under an optical microscope. Those MPs larger than 1 mm were further characterized by FTIR and identified by comparing FTIR spectra with our own database and with open-access databases.^33^

In all samples measured, there was a concentration of MPs within the 820–1340 fragments kg^–^^1^ of dry sample concentration range and Figure 3 shows the distribution of the concentration of MPs observed as a function of the particle size, which varied from ≤450 to ≥2000 μm. As expected, and in agreement with previous studies,^24,40–42^ the results obtained suggest that MPs are further fragmented during composting. As can be observed in Figure 3, the number of larger fragments (≥2000 μm) observed in substrate was 800 ± 400 fragments kg^–1^, which is significantly reduced in both compost samples having 444 ± 260 and 400 ± 150 fragments kg^–1^ in compost 1 and compost 2, respectively. Besides, fragments with sizes between 450 and 2000 μm were not observed in substrate but were present in both compost 1 and compost 2, which can only be attributed to the fragmentation of larger particles during composting. Finally, although further research is needed, it is important to point out that the presence of biochar during composting seems to enhance MP fragmentation, as the lower levels of MP fragments observed in compost 2 could be explained by an increase in the number of fragments not measurable with our method (i.e., extremely small fragments and nanoplastics). Another possible explanation is that the addition of biochar results in higher levels of MP degradation, as observed by Sun and coauthors.^31^ According to these authors, the addition of biochar increased the number of oxygen-containing surface functional groups, leading to higher surface roughness and enhanced hydrophilicity of MPs. Furthermore, biochar addition changed the composition of the microbial community, promoting MP degradation. This could explain the differences observed between compost 2 and compost 1.

**Figure 3:**
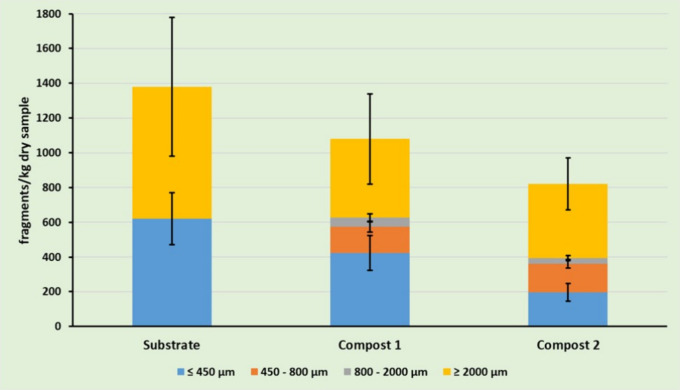
Distribution of microplastics detected in the analyzed samples as a function of the particle size. (Composts 1 and 2 represent compost obtained without and with addition of biochar, respectively).

MP identification by FTIR was considered successful when the matching score, by comparing sample spectra with open-access databases, was higher than 70%, as recommended by the Technical Subgroup on Marine Litter at the European Commission.^35^ It was concluded that three polymers represented the totality of plastic items identified: polyethylene (PE) (including both low and high density), polyethylene terephthalate (PET), and polypropylene (PP) in order of abundance, and the distribution in the different samples (calculated as the ratio between the total number of MPs of a specific type and the total number of isolated MP particles) is shown in Figure 4. As mentioned above, the observed fragmentation during composting prevented the identification of a large fraction of MPs since, although it was possible to observe their presence under the optical microscope, it was not possible to collect them for identification by FTIR. Thus, in both compost samples, only MP fragments of PE were identified, whereas it was not possible to identify PET and PP fragments. As an illustrative example, Figure S1 shows some microplastic fragments detected in the three samples analyzed and the information associated with their identification.

**Figure 4:**
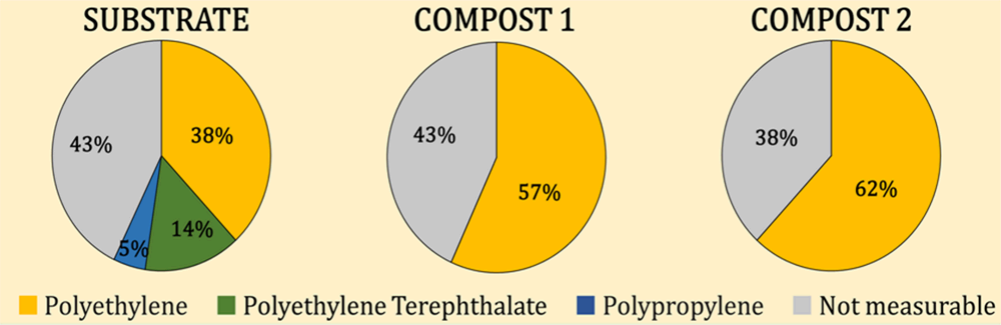
Distribution of microplastics detected and identified by FTIR in the analyzed samples (composts 1 and 2 represent composts obtained without and with addition of biochar, respectively).

### 3.3 Assessment of the Cytotoxicity of Collected Mesoplastics

Previous studies have successfully used *in vitro* cytotoxicity assays to evaluate the effects of plastic pollution, including those related to enhanced bioavailability of microplastic-bound pollutants.^32,43^ In this study, the potential cytotoxicity of the leachable chemicals from plastic debris in the compost was measured. Methanolic extracts of mesoplastics collected from the different samples (substrate, compost 1, and compost 2) and reference plastics (food packaging film, water bottles, and drinking straws) were obtained as described in Section 2 and used for cytotoxicity assessment. It was observed that only extracts from substrate and food packaging film (predominantly PE) displayed cytotoxicity (Figure 5). According to the cytotoxicity data, it is difficult to establish a direct relationship between the toxic effects and the identified MP items (Figure 4). Although all the three samples (substrate, compost 1, and compost 2) contained significant amounts of PE, if PE were responsible for the observed cytotoxicity in substrate, this would also be evident in compost 1 and compost 2. On the other hand, pure polymer controls are required to study the specific contributions of plastic polymer types. However, as this study involved a significant proportion of unidentified plastic polymers (Figure 4), the aim was not to identify the specific polymer that caused cytotoxicity. Moreover, the observed cytotoxic effects could be caused by plastic additives that could have been extracted from both mesoplastics in samples and reference plastics or even by pollutants sorbed to mesoplastics in samples.

**Figure 5:**
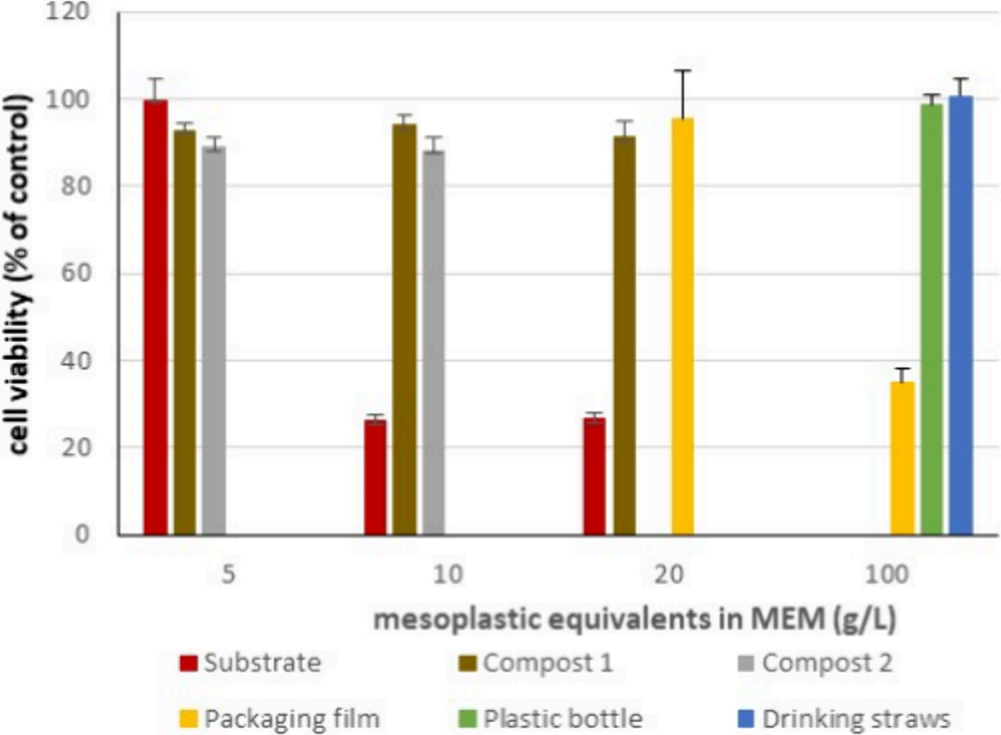
Cytotoxicity observed in extracts from mesoplastics in substrate and compost samples, as well as in reference plastics: packaging film (predominantly polyethylene), plastic bottles predominantly (polyethylene terephthalate), and drinking straws (predominantly polypropylene). Extract concentrations were expressed as grams of mesoplastic equivalents per liter, corresponding to the initial mass of material used for extraction and subsequent dilution in MEM. Cell viability was measured as a percentage of Neutral Red Uptake relative to the growth medium control (supplemented MEM).

As mentioned above, no cytotoxicity was observed in extracts from composts 1 and 2. This suggests that the applied composting process facilitated the elimination of the mesoplastic-associated cytotoxicity, which is consistent with our previous studies showing that the cytotoxicity of organic extracts from compost can be linked to operating conditions.^44^ In this study, we observed that operating conditions influence the reduction of MPs in composts, which is consistent with the findings of other researchers.^45^ However, we also found that these processes could reduce the intrinsic toxicity linked to plastics in organic amendments, thereby minimizing the risk of pollution of nearby aquatic environments by composted soil.

## 4 Conclusions

In the present work, an optimized method was applied to assess the effect of composting and the inclusion of biochar during composting on the presence of microplastics. In parallel, the substrate of both samples was also analyzed for comparative purposes. The estimated concentration of microplastics ranged from 820 to 1340 fragments/kg of dry sample, depending upon the sample, with three polymers (PE, PET, and PP in order of abundance) representing the totality of identified plastic. The results obtained suggested that microplastics are further fragmented during composting and that the presence of biochar could enhance this process. Although further research is needed, these results are concerning, since smaller plastic fragments are more mobile and would therefore be able to reach other environmental compartments more easily.

However, the observed cytotoxicity in the substrate but not in the compost samples could not be linked to the identified plastic items themselves. To achieve this, the use of analytical polymer standards and the whole identification of polymer types is necessary. Instead, the cytotoxicity is likely associated with a combination of factors, including plastic additives and contaminants adsorbed to mesoplastics, which may be coextracted during sample preparation. Furthermore, the absence of cytotoxicity in the mesoplastic extracts of the compost samples suggests that the composting processes were effective in eliminating the compounds responsible for cytotoxicity. In this regard, using ecotoxicological tools, such as the *in vitro* cytotoxicity assay presented here, can provide valuable additional information alongside that produced by quantifying and identifying microplastics. This results in a more comprehensive understanding of their presence and their potential environmental effects.

## Supplementary Material



## Notes

FTIR spectra of the selected microplastic particles (PDF)
