# Supplementary material for: Occurrence of Microplastics and Cytotoxic Effects of Organic Extracts from Isolated Mesoplastics in Compost
Source: ACS Omega. 2026 May 19;11(21):31451–8. doi: 10.1021/acsomega.6c01919 (PMC13234659; doi:10.1021/acsomega.6c01919)

# SUPPLEMENTARY INFORMATION

## OCURRENCE OF MICROPLASTICS AND CYTOTOXIC EFFECTS OF ORGANIC EXTRACTS FROM ISOLATED MESOPLASTICS IN COMPOST

Paloma Sánchez-Argüello[1,*], Gema Sáez-Salto[1], Alice Budai[2], Pierre-Adrien Rivier[2], Simon Weldon[2], Antonio Martín-Esteban[1]

[1] Departamento de Medio Ambiente y Agronomía. INIA-CSIC. Carretera de A Coruña km. 7.5. 28040 Madrid, Spain

[2] Division of Environment and Natural Resources, Norwegian Institute of Bioeconomy Research (NIBIO), Høgskoleveien 7, 1432 Ås, Norway

e-mail: arguello@inia.csic.es; Fax: +34 91 3574008; Tel: +34 91 3478749

*Author to whom correspondence should be addressed.

**Figure S1.-** Example of microplastic fragments detected in the samples analysed and the information associated for its identification by FT-IR.

| | | | |
|---|---|---|---|
| **SUBSTRATE** | **Texture** | Hard | 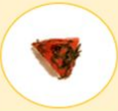 |
| | **Colour** | Red | |
| | **Size** | >2000 µm | |
| | **Composition** | Polypropylene | |

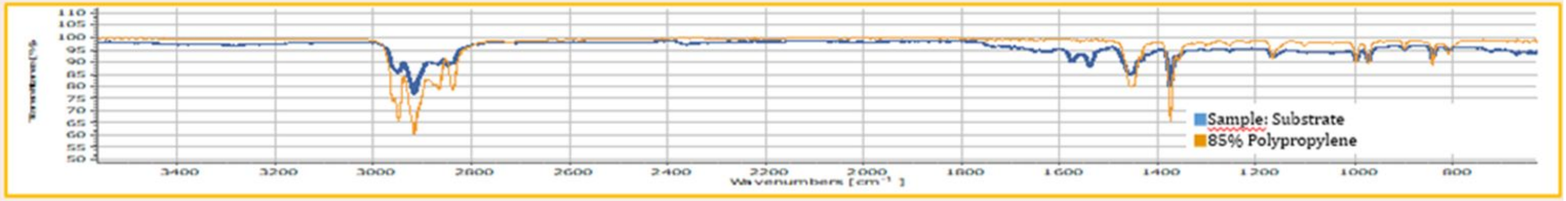


| | | | |
|---|---|---|---|
| **COMPOST 1** | **Texture** | Malleable | 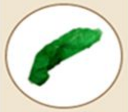 |
| | **Colour** | Green | |
| | **Size** | >2000 µm | |
| | **Composition** | Polyethylene | |

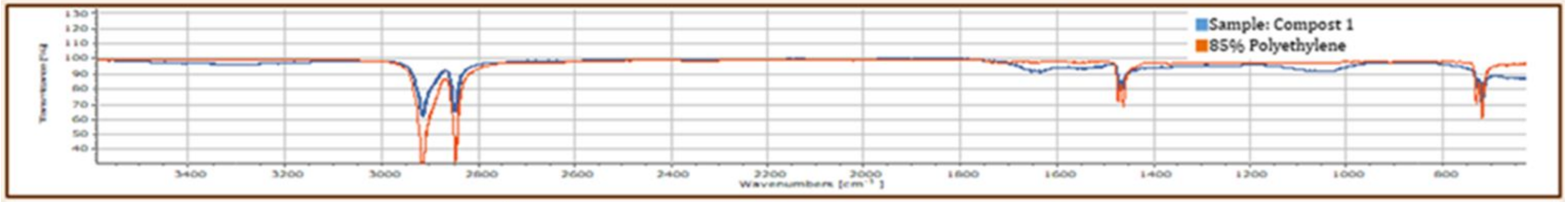


| | | | |
|---|---|---|---|
| **COMPOST 2** | **Texture** | Warpable | 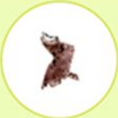 |
| | **Colour** | Purple | |
| | **Size** | >2000 µm | |
| | **Composition** | Polyethylene | |

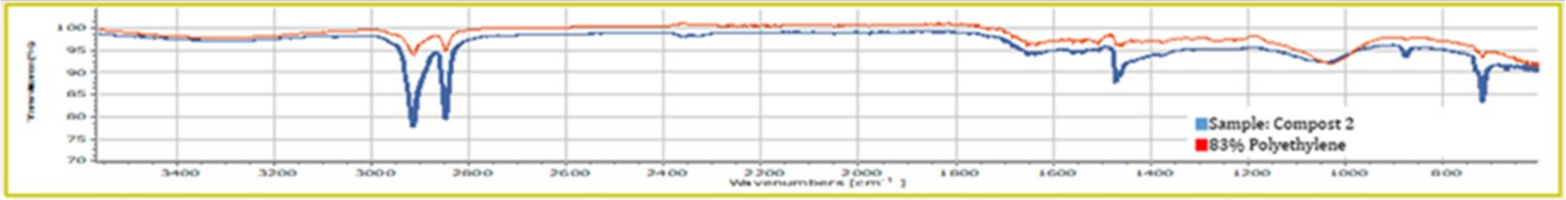

Supplement: Supplementary file 1 [file ao6c01919_si_001.pdf]
